# Poor performance of malaria rapid diagnostic tests for the detection of *Plasmodium malariae* among returned international travellers in China

**DOI:** 10.1186/s12936-023-04596-1

**Published:** 2023-05-24

**Authors:** Jingyao Wu, Jianxia Tang, Weiming Wang, Gangcheng Chen, Xiaoqin He, Sui Xu, Yuanyuan Cao, Yaping Gu, Guoding Zhu, Jun Cao

**Affiliations:** 1 National Health Commission Key Laboratory of Parasitic Disease Control and Prevention, Jiangsu Provincial Key Laboratory on Parasite and Vector Control Technology, Jiangsu Institute of Parasitic Diseases, Wuxi, China; 2 Center for Global Health, School of Public Health, Nanjing Medical University, Nanjing, China

**Keywords:** Malaria, *Plasmodium malariae*, Rapid diagnostic tests (RDTs), Prevention of re-establishment

## Abstract

**Background:**

Malaria is a worldwide infectious disease. For countries that have achieved malaria elimination, the prevention of re-establishment due to infections in returned travellers has become important. The accurate and timely diagnosis of malaria is the key in preventing re-establishment, and malaria rapid diagnostic tests (RDTs) are frequently used due to their convenience. However, the RDT performance in *Plasmodium malariae (P. malariae)* infection diagnosis remains unknown.

**Methods:**

This study analysed epidemiological features and diagnosis patterns of imported *P. malariae* cases from 2013 to 2020 in Jiangsu Province and evaluated the sensitivity of four parasite enzyme lactate dehydrogenase (pLDH)-targeting RDTs (Wondfo, SD BIONLINE, CareStart and BioPerfectus) and one aldolase-targeting RDT(BinaxNOW) for *P. malariae* detection. Furthermore, influential factors were investigated, including parasitaemia load, pLDH concentration and target gene polymorphisms.

**Results:**

The median duration from symptom onset to diagnosis among patients with *P. malariae* infection was 3 days, which was longer than that with *Plasmodium falciparum* (*P. falciparum*) infection. The RDTs had a low detection rate (39/69, 56.5%) among *P. malariae* cases. All tested RDT brands had poor performance in *P. malariae* detection. All the brands except the worst-performing SD BIOLINE, achieved 75% sensitivity only when the parasite density was higher than 5000 parasites/μL. Both pLDH and aldolase showed relatively conserved and low gene polymorphism rates.

**Conclusions:**

The diagnosis of imported *P. malariae* cases was delayed. The RDTs had poor performance in *P. malariae* diagnosis and may threaten the prevention of malaria re-establishment from returned travellers. The improved RDTs or nucleic acid tests for *P. malariae* cases are urgently needed for the detection of imported cases in the future.

## Background

Each year, millions of malaria cases occur worldwide, especially in tropical and subtropical regions [1]. *Plasmodium malariae* is frequently coendemic with *Plasmodium falciparum* in sub-Saharan Africa, South America, Southeast Asia and the western Pacific [2]. The transmission strategy of *P. malariae* is different from that of *P. falciparum*. *Plasmodium malariae* can establish a large parasite reservoir in asymptomatic carriers with low parasitaemia. Although *P. malariae* infection is relatively mild compared with *P. falciparum* infection, it can develop into chronic nephrotic syndrome, which has a high rate of mortality [3].

With the development of international communication and cooperation, the increasing movement of the population between countries leads to a number of travel-related infections, and malaria is one of the main infections diagnosed in African countries and workers [4]. For countries where malaria has been eliminated, the risk of re-establishment via imported malaria cases in populations engaged in overseas work, business, tourism and so on, does exist [5]. For the prevention of re-establishment (POR), rapid and reliable diagnosis is required, and imported malaria cases continue to pose challenges for diagnosis and management in non-endemic countries. The ability to detect imported malaria cases has become the key issue in malaria prevention and control.

Microscopy and rapid diagnostic tests (RDTs) are recommended by the World Health Organization (WHO) for confirmation of diagnosis in suspected malaria patients [1]. Due to their simplicity, cost effectiveness and field readiness, RDTs can provide a quick diagnosis, especially in non-endemic countries where it is difficult to maintain microscopic capabilities and have few imported cases each year; accordingly, RDTs have been used increasingly frequently for malaria diagnosis [6]. However, the sensitivities of RDTs can vary, with unequal sensitivity for different *Plasmodium* species. Reported RDT sensitivity is generally good for *P. falciparum,* but only moderate for *Plasmodium vivax* (66.0–88.0%) [7]. Detection of *P. malariae* and *Plasmodium ovale* (*P. ovale*) using RDTs is less accurate and highly heterogeneous, mainly because of limited sample numbers [8]. Previous study indicated that RDTs had only 5.3–75.4% sensitivity for *P.ovale*, even when the parasitaemia load was larger than 500 parasites/µL [9]. For *P. malariae*, there are even fewer related studies and tested samples.

Considering past malaria epidemic patterns, whether RDTs are useful in diagnosing imported *P. malariae* cases is a major concern. In this study, the characteristics of imported *P. malariae* cases were analysed and the sensitivity of five RDTs in detecting *P. malariae* to identify the ideal diagnostic methods was evaluated, which can be applied in the POR of malaria. Moreover, the factors that influenced the results of RDTs in the detection of *P. malariae* were also investigated.

## Methods

### Epidemiological features of imported malaria cases

Imported malaria cases in China and Jiangsu Province from 2013 to 2020 were analysed. Information on national imported malaria cases was collected from previously published papers. Epidemiological data of imported *P. malariae* cases in Jiangsu Province were collected from the China Information System for Disease Control and Prevention (CISDCP).

The age, sex and origin country distributions of the reported imported malaria cases were collected and analysed, and the duration between patients’ return to China to symptom onset and symptom onset to first medical facility visit were analysed.

### Testing procedures for imported malaria cases

Clinic attendees with fever symptoms were tested for malaria by either microscopy examination or a RDT; both methods were used in medical facilities at the county and above levels. Positive results were reported via the CISDCP within 24 h, and 5 ml venous whole blood was collected for further confirmation. The Center for Disease Control and Prevention in the county where the case was reported rechecked the slides sent by the medical facilities or analysed the collected venous blood sample. Finally, all the whole blood samples were sent to the provincial laboratory within 2 weeks, where all the samples underwent both microscopic examination and nucleic acid-based molecular testing (PCR) [10]. The samples were then kept at − 80 °C, avoiding repeated frozen-thawing.

### Samples collection from *P. malariae* cases

The patients included international travellers and those who worked abroad from 2013 to 2017. Blood samples were collected from malaria patients at local designated hospitals and health centres in Jiangsu Province. Five millilitres of venous blood was collected by venepuncture into an EDTA tube. The collected blood samples were preserved at 4 °C and transported to a provincial malaria diagnostic reference laboratory for reconfirmation. Only *P. malariae*-positive blood samples, confirmed both by microscopy and a nested PCR assay, were included in the study; mixed infection samples were excluded [11].

### Microscopic examination and parasite density determination

The peripheral blood of patients was prepared as thick and thin films. Both smears were stained with 3% Giemsa for 30 min at room temperature. All slides were read under100x oil immersion. Thick films were used for counting parasites. Leucocytes, regarded as 8000/µL, and parasites were used to determine the parasite density [12]. All of the slides were examined by two microscopists, and the results were averaged.

### RDTs for *P. malariae* detection

Five RDTs were evaluated: Wondfo Diagnostic Kit for Malaria (Pf/Pan) (colloidal gold)(Guangzhou Wondfo Biotech Co., Ltd. lot W05481203), SD BIOLINE Malaria Ag Pf/Pan (Standard Diagnostic Inc. Product code 05FK60 lot 05EDC028A), CareStart Malaria Pf/PAN (HRP2/pLDH) Ag Combo RDT (Access Bio, Inc. Product code RMRM-02571 lot MR17M61),BioPerfectus diagnostic kit for malaria (Jiangsu BioPerfectus Technologies Co., Ltd. lot 20180903), and BinaxNOW (Alere Scarborough, Inc. Product code #660-000 lot 097756). The first four brands target Pan-LDH to detect *Plasmodium* spp. BinaxNOW targets Pan-aldolase to detect *Plasmodium* spp. The stored frozen samples were used. Each blood sample was tested by all the five RDTs, with some exceptions due to limited sample volumes. All of the detection procedures followed the manufacturers’ instructions.

### Assessment of pLDH levels in *P. malariae* samples

A quantimal pLDH CELISA kit (Cellabs Pty Ltd, Australia, Product code KM7, lot MPMW26) was used to quantify the density of pLDH in malarial blood samples. The detection principal is sandwich ELISA. The kit uses anti-pLDH capture antibody precoated microwells to bind pLDH in the samples to all *Plasmodium* spp. Then, a labelled anti-pLDH antibody is bound to the complex, allowing a chromogenic reaction. As soon as the stopping solution is added, the colour intensity is proportional to the concentration of pLDH in the sample. Forty-five *P. malariae* samples were tested in this study. All procedures followed the kits’ instructions. The results were read with a spectrophotometer at 450 nm/620 nm.

### *Plasmodium malariae* LDH and aldolase gene sequencing

Genomic DNA was extracted from 200 µL blood samples with a QIAmp Blood Mini Kit (QIAGEN) according to the manufacturer’s instructions. The *P. malariae* aldolase gene was amplified using primers ALf (5′-CAGGCATCAAGCGCAGACTA-3′) and ALr (5′-TAAAGCCCATGGGTGAGGTC-3′) [13], while the *P. malariae* LDH gene was amplified using primers LDHf (5'-ACTTTACAGCCGCCCATTCC-3') and LDHr (5′-CCTTCATTCTCTTCGTTTCAGCA-3′) [14]. Conventional PCRs were conducted in 20 µl volumes with a KOD-401 kit. The products were sequenced by Shanghai exsyn-bio Technology Co., Ltd., and the sequence results were analysed with MEGAX.

### Statistical analysis

All the results were analysed using SPSS 19.0 and GraphPad Prism 8.3.0. Mann–Whitney test was used to analyse the variance of epidemiological data. The sensitivity of each brand as well as the sensitivity of each brand at different parasite density levels were assessed. The sensitivities of the 4 LDH-targeting brands at different pLDH concentration levels were calculated. Uncertainty was interpreted with 95% confidence intervals (CIs). Categorical variables were determined by Chi-squared tests. Fisher’s exact correction was applied when the expected frequency in the cell was 5 or less. Pearson’s correlation analysis was used to analyse correlation between parasite density and pLDH OD level. All the *p* value < 0.05 were considered as significant.

## Results

### Delayed diagnosis and misdiagnosis of imported *P. malariae* cases

**Fig. 1:**
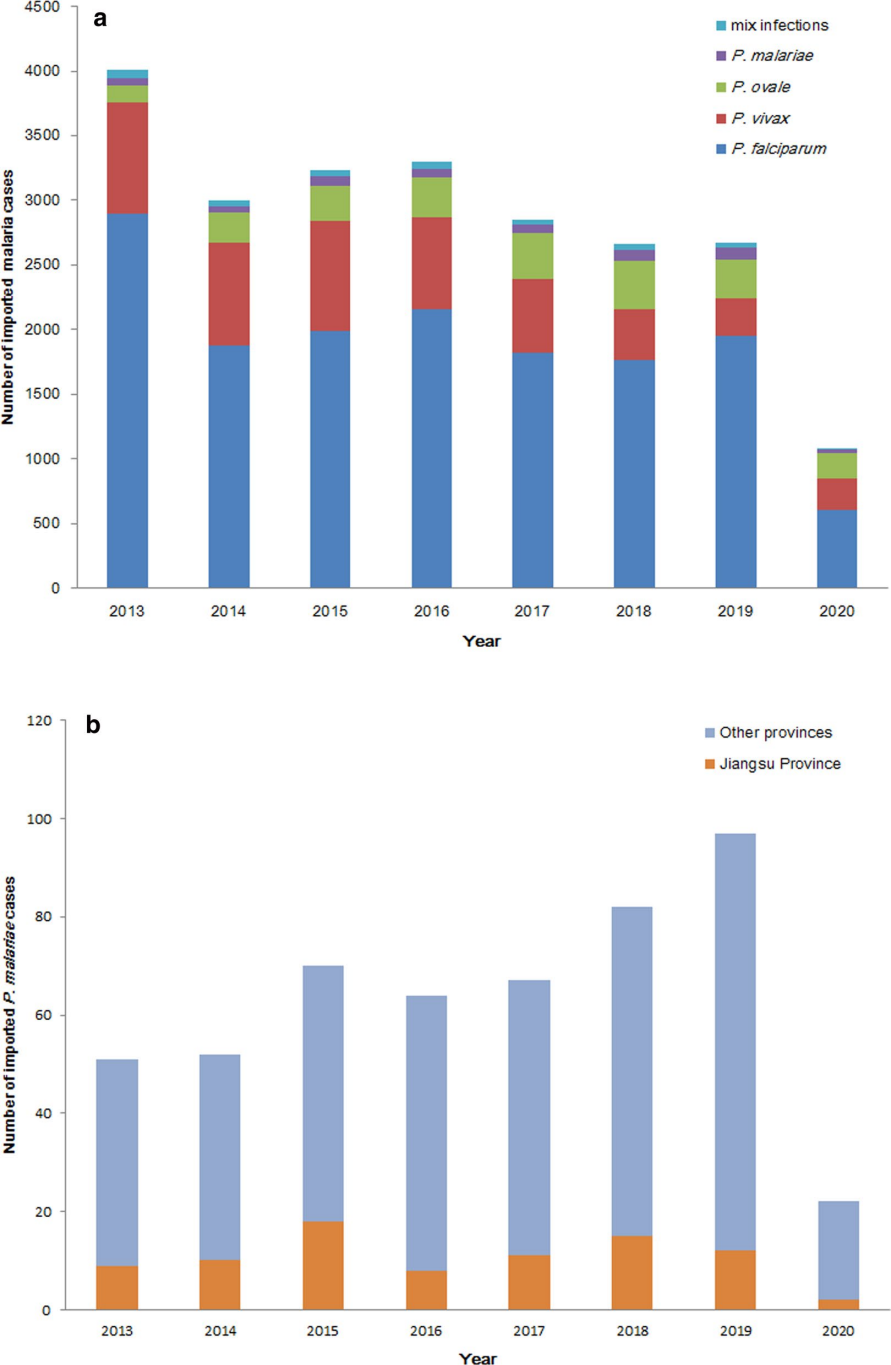
Number of imported malaria cases in China. **a** Number of imported malaria cases in China, 2013–2020, **b** Number of imported *P. malariae* cases in China, 2013–2020

From 2013 to 2020, 505 imported *P. malariae* cases at the national level in China and 85 cases in Jiangsu Province were diagnosed. During the 8-year period, there was a general increase in the number of *P. malariae* cases at the national level, from 51 cases in 2013 to 97 cases in 2019. Due to the decreased travel caused by coronavirus disease 2019 (COVID-19), the number of imported *P. malariae* cases in China decreased to 22. Among these, there were more than 10 imported *P. malariae* cases nearly every year in Jiangsu Province from 2013 to 2019 (Fig. 1a, b) [15–20].

**Fig. 2:**
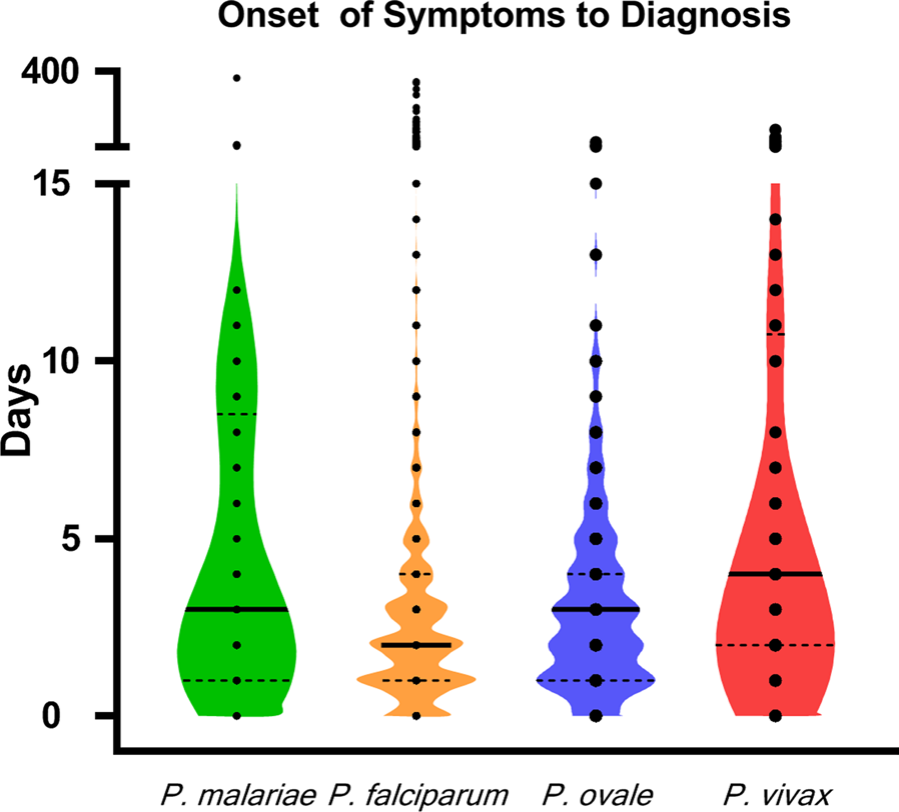
Comparison of the time from symptom onset to diagnosis in Jiangsu Province, 2013–2020. The middle line of each violin plot represents the median, while the dotted line represents the interquartile range. The black dots represent individual cases

The majority of *P. malariae* infection patients in 2013–2020 in Jiangsu Province were males (66/69, 95.7%) and aged 41–50 years (31/69, 44.9%). All of the cases originated from Africa, among which Angola accounted for the most cases (19/69, 27.5%), followed by Equatorial Guinea (15/69, 21.7%) and Nigeria (6/69, 8.7%) (Table 1). The duration from symptom onset to *P. malariae* diagnosis was different from that to *P. falciparum* diagnosis (U = 47024, P = 0.0015 < 0.05), and the median for *P. malariae* was 3 days (interquartile range, IQR: 1–9), which was longer than 2 days (IQR: 1–4) for *P. falciparum* (Fig. 2). The duration from returning from a malaria-endemic country to the onset of malaria symptoms was more than 50 days in 17 cases (24.6%, 17/69), among which 6(8.7%) had durations of more than 100 days. The median duration from symptom onset to the first medical facility visit was 2 days (IQR: 0–4). The median time for medical facility to make medical diagnosis was 0 days (IQR: 0–1). Sixty-one percent (42/69) of the patients were diagnosed at the first medical facility visit, 24.6% (17/69) were diagnosed 3 days later, and 14.5% (10/69) were diagnosed more than 3 days later. The longest diagnosis time was 27 days.

**Table 1:** The characteristics of imported *P. malariae* cases in Jiangsu Province, China, 2013–2020

	Number of cases n = 69 (%)
Sex	
Male	66 (95.7)
Female	3 (4.3)
Age	
≤ 30	10 (14.5)
31–40	19 (27.5)
41–50	31 (44.9)
> 50	9 (13.1)
Origin of infection	
Angola	19 (27.5)
Equatorial Guinea	15 (21.7)
Nigeria	6 (8.7)
Congo (Brazzaville)	5 (7.2)
Congo (Kinshasa)	5 (7.2)
Liberia	4 (5.8)
Gabon	4 (5.8)
Cameroon	4 (5.8)
Republic of the Sudan	2 (2.9)
Chad	1 (1.4)
Zambia	1 (1.4)
Mozambique	1 (1.4)
Togo	1 (1.4)
Kenya	1 (1.4)
Parasitaemia load (p/μL)	
< = 1000	16 (23.2)
1001–5000	39 (56.5)
> = 5001	14 (20.3)

All 69 *P. malariae* cases were detected at and reported by county- or above-level facilities, and both microscopic examinations and RDTs were used. However, among the analysed cases, 30 of 69 cases (43.5%) were misdiagnosed as negative via RDTs (Wondfo Diagnostic Kit for Malaria (Pf/Pan) (colloidal gold)) at the county level which exhibited a relatively low detection rate.

### Poor performance of the five RDTs in *P. malariae* detection

In total, 45 *P. malariae* monoinfection cases from 2013 to 2017 were included in the study. The performances of the five RDTs (Wondfo, SD BIOLINE, CareStart, BinaxNow, BioPerfectus) were compared with regard to *P. malariae* detection. Each sample was tested using all five brands, with some exceptions due to limited sample volumes. The results indicated that CareStart test had the highest sensitivity, at 72.7% (95% CI 59.0–86.4%), while the SD BIOLINE test had the lowest sensitivity, at 13.3% (95% CI 3.0–23.7%) (Table 2). Overall, all five RDTs exhibited relatively low sensitivity for the detection of *P. malariae*.

**Table 2:** Sensitivities of the five RDTs for the detection of *P. malariae* monoinfection

Brand	No. of samples	No. of positive samples	No. of negative samples	Sensitivity (%) (95% CI)
Wondfo	45	28	17	62.2 (47.5 ~ 77.0)
SD BIOLINE	45	6	39	13.3 (3.0 ~ 23.7)
CareStar	44	32	12	72.7 (59.0 ~ 86.4)
BinaxNOW	44	29	15	65.9 (51.3 ~ 80.5)
BioPerfectus	43	26	17	60.5 (45.2 ~ 75.7)

CI, 95% confidence interval

### Poor performance of the five RDTs in patients with low parasitaemia

Each RDT result was evaluated according to the parasitaemia load of the samples. According to the parasite density, samples were divided into 3 groups (≤ 1000, 1001–5000 and ≥ 5001 parasites/µL). Sensitivity was calculated based on the performance of each brand in each group. The Wondfo, CareStart, BinaxNOW and BioPerfectus tests had sensitivities of 75%, 83.3%, 83.3% and 75%, respectively, when parasite densities were higher than 5000 parasites/µL. The categorical variable parasite densities were analysed and showed a result of no significance (Fisher’s exact = 2.00, *P* = 0.36 for Wondfo; Fisher’s exact = 1.01, *P* = 0.67 for CareStart; Fisher’s exact = 5.54, *P* = 0.06 for BinaxNOW; Fisher’s exact = 27.50, *P* = 0.263 for BioPerfectus). The SD BIOLINE test had only 41.7% sensitivity, even at parasite densities above 5001 parasites/μL (Table 3). The categorical variable analysis showed significance (Fisher’s exact = 8.43, *P* < 0.05). All four RDTs exhibited poor performance (sensitivities from 33.3 to 66.7%) when parasite density was low (≤ 1000 parasites/μL), while the SD BIOLINE test could not detect any *P. malariae* when the parasite density was lower than 1000 parasites/μL (Table 3).

**Table 3:** Comparison of the five RDTs for the detection of *P.malariae* categorized by parasite density

Parasite density (parasites/µL)	No. of samples	No. of positive samples	Sensitivity(%) (95%CI)
Wondfo			
≤ 1000	9	4	44.4 (3.9 ~ 85.0)
1001–5000	24	15	62.5 (41.6 ~ 83.4)
≥ 5001	12	9	75.0 (41.1 ~ 100)
SD BIOLINE			
≤ 1000	9	0	0.0 (0 ~ 0)
1001–5000	24	1	4.2 (0 ~ 14.2)
≥ 5001	12	5	41.7 (8.9 ~ 74.4)
CareStart			
≤ 1000	9	6	66.7 (20.8 ~ 100)
1001–5000	23	16	69.6 (49.2 ~ 89.9)
≥ 5001	12	10	83.3 (54.2 ~ 100)
BinaxNOW			
≤ 1000	9	3	33.3 (0 ~ 79.2)
1001–5000	23	16	69.6 (49.2 ~ 89.9)
≥ 5001	12	10	83.3 (54.2 ~ 100)
BioPerfectus			
≤ 1000	8	3	37.5 (0 ~ 89.5)
1001–5000	23	14	60.9 (39.3 ~ 82.4)
≥ 5001	12	9	75.0 (41.1 ~ 100)

CI, 95% confidence interval

### Poor performance of the four LDH-targeting RDTs under low-pLDH concentration conditions

The pLDH concentrations in the samples were quantified through ELISA test. The results were presented as optical density (OD) values. To correlate pLDH concentrations with the sensitivities of the four pLDH-based RDTs, 45 samples were divided into three groups (≤ 0.150, 0.151–1.500 and ≥ 1.501) according to their pLDH concentrations (Table 4). When the OD value of the pLDH concentration reached 1.5, the Wondfo, CareStart and BioPerfectus tests had 100% sensitivity. The SD BIOLINE test had a much lower sensitivity (27.3%), even when the pLDH concentration was over 1.5 (Table 4). The categorical variable pLDH concentrations were analysed and showed a result of significance (Fisher’s exact = 39.04, *P* < 0.05 for Wondfo; Fisher’s exact = 33.06, *P* < 0.05 for CareStart; Fisher’s exact = 39.04,* P* < 0.05 for BioPerfectus; Fisher’s exact = 5.36, *P* < 0.05 for SD BIOLINE).

**Table 4:** Comparison of the four RDTs for the detection of *P. malariae* categorized by pLDH concentration

pLDH concentration (OD)	No. of samples	No. of positive samples	Sensitivity(%)(95%CI)
Wondfo			
≤ 0.150	12	0	0 (0 ~ 0)
0.151–1.500	9	4	44.4 (3.9 ~ 85)
≥ 1.501	22	22	100 (100 ~ 100)
SD			
≤ 0.150	12	0	0 (0 ~ 0)
0.151–1.500	9	0	0 (0 ~ 0)
≥ 1.501	22	6	27.3 (7.1 ~ 47.5)
CareStart			
≤ 0.150	12	1	8.3 (0 ~ 29.9)
0.151–1.500	9	8	88.9 (58.3 ~ 100)
≥ 1.501	22	22	100 (100 ~ 100)
BioPerfectus			
≤ 0.150	12	0	0 (0 ~ 0)
0.151–1.500	9	4	44.4 (3.9 ~ 85)
≥ 1.501	22	22	100 (100 ~ 100)

CI, 95% confidence interval

### A moderate correlation between the pLDH concentration and parasitaemia load

**Fig. 3:**
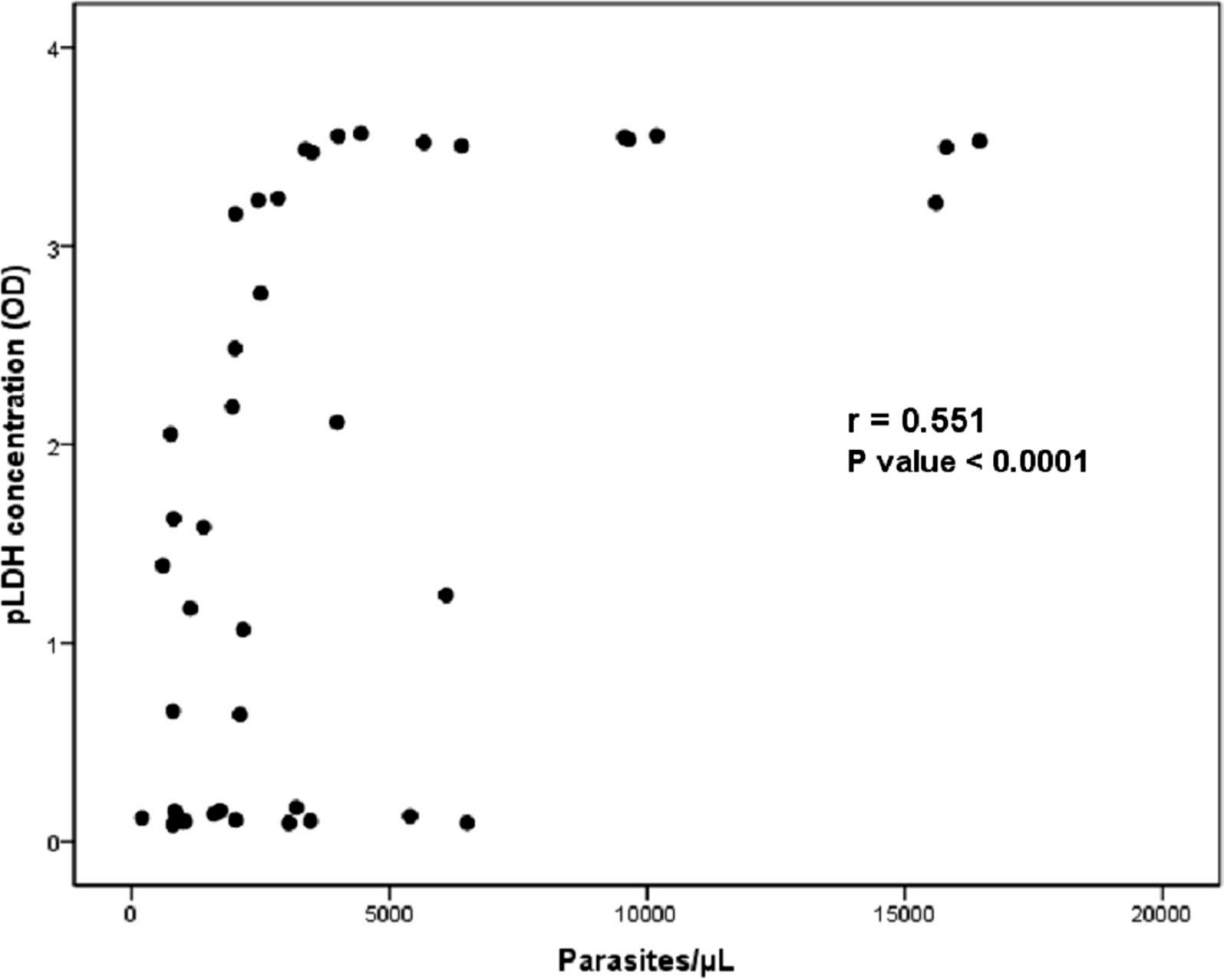
Correlation between parasite density and pLDH concentration among samples

The correlation between pLDH concentration and parasite density was evaluated to assess whether the pLDH concentrations in samples were associated with the samples’ parasite densities (Fig. 3). The r value was 0.551 (*P* < 0.0001), which represents a moderate correlation between the two factors.

### Relatively conserved pLDH and aldolase sequences among *P. malariae* samples

pLDH and aldolase sequences from samples were obtained, and the results were analysed with MEGAX. Amplification failed in some low-parasitaemia samples. However, the PCR products of the *P. malariae* LDH gene from 39 samples yielded 982 base pairs, and the *P. malariae* aldolase fragments from 39 samples yielded 912 base pairs. No nucleotide substitutions were observed in the LDH gene of *P. malariae* compared to the reference sequence (NCBI Reference Sequence: XM_029006607.1). Three nucleotide substitutions were observed in the aldolase gene of *P. malariae* compared to the reference sequence (NCBI Reference Sequence: XM_029006963.1). Among the substitutions, 2 were synonymous mutations, and 1 was a nonsynonymous substitute with T681A in 4 samples.

## Discussion

There have been thousands of imported malaria cases annually since 2013 in China; *P. malariae* cases have been imported every year and have shown an increasing trend in recent years. The imported *P. malariae* cases all originated from Africa and mainly occurred in males, which coincided with previous findings that most of the imported malaria cases in China occurred in workers returning from Africa and other malaria-endemic areas [21]. These *P. malariae* cases had longer durations from symptom onset to diagnosis than falciparum cases. These findings may attract attention because the longer the time for patients to be diagnosed after symptom onset is, the higher the risk of severe symptoms is. It also increases the risk of introducing malaria locally, posing a challenge in the prevention of malaria re-establishment.

The WHO recommends that all suspected malaria cases receive parasitological confirmation before drugs administered. Microscopic examination used to be the most popular detection method for malaria parasites. In recent years, RDTs have been recommended by the WHO to assist in the rapid and accurate diagnosis of malaria and have been gradually increasingly applied, especially in the preliminary screening of suspected cases. It could be predicted that in non-endemic areas, which may face challenges in maintaining microscopic examination capability, RDTs will be used more widely and even replace microscopic examinations at the local level. However, according to the 69 analysed *P. malariae* cases, the false-negative rate of RDTs was 43.5% at the local level. All five RDTs we tested showed poor performance for *P. malariae* detection, with relatively low sensitivity. To date, the WHO has not recommended criteria for *P. malariae* testing. Moreover, by using the same sensitivity thresholds for determining whether a RDT adequate detects *P. falciparum* and *P. vivax*, all the tested RDT sensitivities in this study for *P. malariae* are inadequate.

In this study, the sensitivity of Wondfo, CareStart, BinaxNOW and BioPerfectus tests were not statistically increased with an increasing parasite density. All of the tests reached more than 70% once the parasite density was more than 5001 parasites/µL, with the exception of the SD BIOLINE test. The four (excluding the BinaxNOW test, which targets Pan-aldolase) Pan-LDH-targeting tests had a different sensitivity with an increasing concentration of pLDH. The sensitivities of all the tests except the SD BIOLINE test reached 100% once the concentration of pLDH was more than 1.50 OD. However, a higher parasitaemia load was not correlated with a higher pLDH concentration; only a moderate correlation was observed between the pLDH concentration and parasite density in the *P. malariae* blood samples, and similar results were also observed in *P. vivax* and *P. ovale* samples and in the rodent model for malaria [9, 22, 23]. In this study, to avoid potential effects of blood sample storage, all blood samples collected in either clinics or hospitals were transported to the provincial laboratory as quickly as possible after species confirmation. Since the blood samples from *P. malariae* infection patients might have contained parasites at different developmental stages, the pLDH concentration instability during the metabolic process in *Plasmodium* parasites may have been the reason for the moderate correlation [22, 24, 25]. The frozen-thawing process of the blood samples may also affect the stability of pLDH, though there are limited literatures on this topic. Only one non-synonymous substitution (T681A) in the tested samples was detected in the aldolase gene sequences, and no nucleotide substitutions were observed in the LDH gene. Both exhibited a relatively conserved and low gene polymorphism rate, suggesting that other reasons may contribute to the poor performance of the five RDTs in *P. malariae* detection.

China was officially awarded the national malaria-free certification from the WHO on June 30, 2021. However, the risk of the re-establishment of malaria from imported cases remains a concern due to increasing international trade and global exchanges [26]. Historically, there were many *P. malariae-*endemic areas with thousands of cases annually, Jiangsu Province included [27]. Although the goal of malaria elimination has been achieved in China, suitable transmission vectors are still widely distributed throughout the country. As a result, *P. malariae* cases among travellers without early diagnosis and appropriate treatment influence the risk of re-establishment, which has been observed in many other countries where malaria has been eliminated for many years such as Armenia [28]. In addition, although *P. malariae* usually causes less severe disease in humans than other forms of malaria, it can still cause chronic nephrotic syndrome, which may lead to death [29, 30]. Unfortunately, all five commonly used RDTs cannot detect *P. malariae* cases efficiently and accurately, which increases the risk of severe symptoms and introduced malaria, posing a challenge to the malaria re-establishment prevention.

Furthermore, for countries in Africa, southeast Asia and other regions with *P. malariae* endemic situation, inefficient and inaccurate RDTs may increase the missed or delayed diagnosis on *P. malariae*, which leads to the adverse clinic consequences even the secondary transmission. Therefore, the diagnosis of *P. malariae* cases must attract more attention, and more advanced RDT products or other methods, such as nucleic acid-based molecular tests, should be developed and adopted to overcome this problem.

## Data Availability

All data generated or analysed during this study are included in this published article.
